# Impact of Diabetic Retinopathy on Health-related Quality of Life in Iranian Diabetics

**Published:** 2017-01

**Authors:** Cyrus ALINIA, Seyed-Farzad MOHAMMADI, Alireza LASHAY, Arash RASHIDIAN

**Affiliations:** 1. Dept. of Health Management and Economics, School of Public Health, Tehran University of Medical Sciences, Tehran, Iran; 2. Eye Research Center, Farabi Eye Hospital, Tehran University of Medical Sciences, Tehran, Iran

**Keywords:** Diabetic retinopathy, Macular edema, Time trade off

## Abstract

**Background:**

To extract utility values of diabetic retinopathy on a perfect health and perfect vision scales for Iranians with both types diabetes.

**Methods:**

In this investigation, 150 untreated patients with diabetic retinopathy consecutively were examined and interviewed in Farabi Eye Hospital, Tehran, Iran, a tertiary referral center in the Iranian health care system. Utility values based on patients-reported outcome measures, standard gamble, time trade off and visual analogue scale approaches, were estimated.

**Results:**

Considering all three utility elicitation methods were valid, diabetic retinopathy patients, on average reported the 0.95 (±0.03), 0.85 (±0.15) and 0.80 (±0.30) standard policy scale utility according to standard gamble, TTO and VAT respectively. In all three-studied approach, diabetic retinopathy had more disutility in higher levels of disease and had more disutility in the presence of both maculopathy and vasculopathy compared with one of them. Evidence show that share of macular edema in imposing disutility was maximum in early stage and exponentially decreased with advancing the severity of diabetic retinopathy.

**Conclusion:**

Study indicated utility scores in DR-experienced Iranian patients were highest with the SG and lowest with the VAT method. The proffered utility-elicitation method in Iranian patient populations could be the TTO approach. The novel model we employed for DR takes the effects of diabetic macular edema and vascularization into account separately, and can provide a better estimate of the QoL for these patients.

## Introduction

Diabetic retinopathy (DR) is an ocular manifestation and serious microvascular complication of diabetes mellitus that often affects working-aged adults (1). Almost all patients with type I diabetes and more than 60% of type II diabetics will have some degree of retinopathy 15–20 years after diagnosis. In developed countries, diabetic eye disease represents the leading reason for visual impairment in adults less than 75 years (2). In Iran, DR prevalence has reached 24–37% among diabetics (3).

Visual impairment because of DR has a significant negative impact on patients’ life span and their quality of life (QoL) (4). QoL is an abstract concept, which exists only in the mind of the person, whose life is affected, and intrinsically, it cannot be directly observed; instead, an individual’s preferences should be elicited (5). Preferences can be measured using either direct disease-specific Standard Gamble (SG) or Time Trade-Off (TTO) instruments, whereby patients themselves value their own health state (6) or indirect multi-attribute ones that attach societal preferences derived from the general population to the health states of patients using general questionnaires, such as the EuroQol-5D (EQ-5D), Short Form-6D (SF-6D) and Health Utility Index (HUI) (7). SG and TTO are two choice-based methods with incontestable reliability, acceptability, and validity in Western sociocultural contexts, valuing the weight of any health state on a numeric scale from 0.00 (death) to 1.00 (perfect quality of life) (8). Economists refer to these values as the utility; a cardinal function also known as the “policy scale” due to its application in value-based policy-making (9). Both SG and TTO have established theoretical underpinnings. The TTO has been developed to be an easy alternative for the SG with empirical properties similar to the standard gamble and overcomes the problems of explaining probabilities to respondents (10). Another less popular direct instrument to elicit utility is the Visual Analogue Scale (VAS) which is a type of rating scale that asks a respondent to indicate his or her current health state on a scale with finish points of death (0) and ideal health (100) (11).

With the advent of new methods for cost-effectiveness evaluation in the 1970s and 1980s, utility assessment techniques have frequently been used to quantify the impact of medical interventions (12). One effectiveness criterion is the Quality Adjusted Life Year (QALY), which is a combination of the quantity and quality of obtained life years attributable to an intervention (13).

In this perspective, for the first time in a middle-income country, we aimed to measure the impact of DR along its spectrum and its clinical pictures on patients’ health-related QoL (HRQoL) using the single-attribute Standard Gamble, TTO, and VAS based on the policy and the perfect vision scales.

## Methods

### Patient Selection

During the winter of 2015, patients were recruited consecutively from those visited at Farabi Eye Hospital, a tertiary referral hospital in the Iranian health care system. Eligible subjects were Persian-speaking Iranians at least 22 yr of age who had diabetes with some degree of untreated DR, were willing and able to participate and lived independently. Patients with Alzheimer’s or another form of dementia were not included. For those who for any reason such as significant hearing or cognitive problems were not able to speak or understand our questions and scenarios, we asked their first-degree relatives as their proxy.

### Data collection

#### Examinations

For all participants, visual acuity was tested by an optometrist using the standard Snellen chart, and they were examined by a senior vitreoretinal subspecialist at the Retina Consultation Unit to assess DR. The macular and optic disc were viewed at the slit lamp using a 90 diopter wide-field lens (Volk Inc., O’Fallon, MO) or Goldman 3 mirror (Volk), if needed, and imaged by fluorescein angiography and optical coherence tomography (OCT) (Heidelberg Engineering, HeidelbergGermany) to evaluate DME.

#### Utility Elicitation

To derive DR utility, face-to-face interview sessions with participants were organized on an individual basis. All interviews were managed and performed by a Persian-speaking health economist. We used direct single-attribute and disease- specific preference-based methods including the SG and TTO in addition to the VAS in both the policy scale and the perfect vision scale for extracting the actual and experienced DR scenarios in different levels of vision health states. The Visual Analogue Thermometer (VAT) instrument was used instead of the VAS in order to reduce its limitations. To apply the SG and TTO techniques, we used the standard protocol modified by Brown et al. (online appendix 1) with 20 years remaining life for conveying the concepts to patients in the scenarios. This adjustment was applied after 12 pilot interviews. To minimize any cognitive biases and considering the subject’s characteristics and the difficulty to understand some questions, we used the following techniques in addition to modifying the scenarios: using a single interviewer for all interviews, repeating the questions several times if needed, and asking proxies when patients could not respond properly.

In the TTO technique, we used the following formula to measure the utility value: 1.0 - (time traded in years/20). In addition, the SG utility was calculated as follows: 1.0 - (risk of death or binocular blindness a patient is willing to accept). SG and TTO scores range from 0.0 (death or binocular blindness) to 1.0 (perfect health or perfect binocular vision) (4). In the VAT method, participants were asked to rate a state along a continuum from 0 (representing “dead/double blindness”) to 100 (representing “perfect health/perfect vision health”). (5). in all methods, 1- utility scores show us disutility of diseases and theirs related divisions.

#### Definitions

Using the better Seeing Eye, we categorized the severity of DR using the patients’ perception of their disease. For DR patients, HRQoL is only affected or significantly changed after tangible changes in visual acuity occur. In other words, patients realize their diseases and its limitations on their QoL in stages of neovascularization and macular edema. Therefore, patient perception should be used instead of other categorizations like definitions used in the Early Treatment Diabetic Retinopathy Study (ETDRS), or other similar studies (15–17). because these classifications are based on the natural course, clinical diagnosis, and follow-up management of DR and not patients’ perception of their vision health. We considered diabetic macular edema (DME) and DR as two parallel sequelae of diabetes that simultaneously affect QoL. Thus, we used the three following vision states in two categories with and without DME: none to moderate NPDR (levels 10–41), severe and very severe NPDR (levels 41–51), and early to high-risk (levels 60–80). To determine the presence of DME, we applied the American Academy of Ophthalmology classification as no DME up to 10/20 and having DME higher than 10/20.

Uncorrected distance monocular and binocular visual acuity were assessed, and visual acuity in the better-seeing eye was categorized based on the International Classification for Visual Impairment as normal (>0.5), mild impairment (<0.33–0.5) moderate impairment (<0.1–0.33), blindness (<0.05–0.1), and very severe to total blindness for cases with visual acuity less than 0.05 (18).

Since almost all participants were elderly, we recorded self-reported major non-diabetic comorbidities such as cancer and myocardial infarction that can theoretically influence HRQoL and inquired about any history of hospitalization in the coronary care unit. We also considered certain diabetic comorbidities that could have a synergic effect on patient’s disutility such as diabetic gastroparesis, neuropathy, nephropathy and diabetic foot, as well as cataract and dry eye. Other recorded information included smoking status, diabetes duration, body mass index (BMI; kg/m2), blood pressure, and the presence of hyperlipidemia. To determine the type of diabetes, we asked about the age of onset and their treatment types (oral or injected hypoglycemic agents). BMI was categorized using the WHO standard groupings (14). To assess relationships between socioeconomic factors and extracted utility, we addressed the participants’ gender, age, marital status, employment status, education level, and per capita monthly income by interviewing and completing a closed questionnaire. To extract the family’s income status, we used the total monthly expenditure as a proxy in three categories of low (below 570 USD), middle (570–1430 USD), and high income (over 1430 USD).

#### Data Analysis

To ensure having at least 20 individuals in each of the three defined states of DR with an effect size of 0.3 for utility scores, a power of 0.8, and a two-tailed significance level of 0.05, a sample size of 150 subjects seemed appropriate.

To assess DR utility, the EXCEL software (Microsoft Corporation, Redmond, WA, USA) was used. The non-parametric Kolmogorov-Smirnov test was used to determine variable normality. Normal variables were summarized as mean and standard deviation (SD), and we determined the median and interquartile range (IQR) if distributions were skewed. For inter-group analyses, we used an independent heteroscedastic-test or within subjects ANOVA test. Differences in mean utility among standard gamble, TTO, and VAT techniques were assessed using paired student *t-*tests. To compare proportion differences, we applied the Chi-squared statistical test.

The stability and reliability of standard gamble, TTO, and VAT methods over time was evaluated by calculating intraclass correlation coefficients (ICC) (19) among 20 patients and after 2 weeks in all DR states.

## Results

Of 158 approached DR patients, 150 participants in 3 vision states completed the study (response rate=94.9%). The median age of the participants was 58 years (range, 22 to 86 years), 79 of them (52.7%) were men, and 92% were urban residents. Of the subjects, 80.7% and 12% were married and single, respectively, and the rest were divorced or widowed. The middle-income group comprised 71.3% of the sample (mean: 895 USD), and 20.3% and 8% were low (mean: 415 USD) and high (mean: 1830 USD) income, respectively. The recorded education level was less than 12 years of elementary and secondary schooling for 75.3% of the participants.

Therefore, the distributions of utility scores obtained from dependent variables’ were positively skewed and significantly differed from normal; this was also confirmed by normality tests. Predictor variables, excluding occupation and visual impairment, were non-normally distributed as well.

### 

#### Study Reliability

The range of test–retest reliability was 0.58–0.77 for SG 0.52–0.70 for TTO and 0.48–0.66 for VAT. This shows acceptable medium to good agreement ranks and confirms the reliability and stability of our methods. The highest agreement coefficient was observed in patients with mild to moderate DR and acceptable lowest ICC was attributed to end-stage DR. On average, the SG approach had relatively lower variance and thus better reliability (Table 1).

**Table 1:** Test-retest reliability for the utility assessment methods in patients with different levels of diabetic retinopathy

**DR States Scenarios**	**SG (n=20)**		**TTO (n=20)**		**VAT (n=20)**	
	*ICC*	*P-value*	*ICC*	*P-value*	*ICC*	*P-value*
No DR (3)	0.61	0.041	0.52	0.098	0.55	0.089
Mild NPDR (5)	0.77	0.011	0.69	0.020	0.64	0.038
Moderate NPDR (5)	0.72	0.025	0.70	0.017	0.66	0.033
Severe NPDR (4)	0.55	0.089	0.54	0.092	0.48	0.102
PDR (3)	0.58	0.071	0.50	0.091	0.59	0.069

DR = diabeticretinopathy; ICC = intraclasscorrelation; NPDR = non-proliferativediabeticretinopathy; PDR = proliferativediabeticretinopathy; SG= standard gamble; TTO = time trade off; VAT = visual analogue thermometer

#### Sociodemographic and Utility

On average, the self-reported utility values in DR-experienced patients were 0.95, 0.85 and 0.80 with the SG, TTOand VAT methods, respectively. Study subjects reported relatively different utility scores based on their sociodemographic status, but differences were not statistically significant. Only TTO and VAT approaches showed the higher significant general disutility attributed to DR in 0.10 significant level on the women and unemployment Iranian diabetics respectively (Table 2).

**Table 2:** Total health/death- utility for all untreated diabetic retinopathy states with the utility assessment methods by sociodemographic factors

**Variable**	**n**	**SG Utility**		**TTO Utility**		**VAT Utility**	
		**Median (IQR)**	***P*-value**	**Median (IQR)**	***P*-value**	**Median (IQR)**	***P*-value**
All	150	0.95 (0.03)		0.85 (0.15)		0.80 (0.30)	
Sex							
Male	79	0.96 (0.15)	0.43	0.89 (0.28)	0.06	0.80 (0.25)	0.26
Female	71	0.94 (0.16)		0.80 (0.30)		0.75 (0.29)	
Age (yr)							
22–35	12	1.00 (0.05)	0.29	0.87 (0.12)	0.14	0.80 (0.14)	0.29
35–50	52	0.95 (0.10)		0.90 (0.30)		0.80 (0.23)	
>50	86	0.93 (0.18)		0.80 (0.30)		0.73 (0.30)	
Marital Status							
Single	18	0.94 (0.10)	0.59	0.80 (0.20)	0.75	0.80 (0.20)	0.57
Married	121	0.95 (0.15)		0.85 (0.30)		0.80 (0.30)	
Other	11	0.90 (0.24)		0.90 (0.13)		0.70 (0.35)	
Occupation Status							
Employed	56	0.96 (0.11)	0.18	0.90 (0.25)	0.23	0.80 (0.21)	0.06
Unemployed	25	0.96 (0.16)		0.80 (0.23)		0.75 (0.25)	
House keeper	47	0.95 (0.12)		0.80 (0.30)		0.80 (0.23)	
Others	22	0.90 (0.21)		0.85 (0.28)		0.65 (0.27)	
Income							
Low	31	0.95 (0.16)	0.91	0.80 (0.23)	0.21	0.72 (0.27)	0.33
Middle	107	0.95 (0.15)		0.90 (0.30)		0.80 (0.30)	
High	12	0.93 (0.13)		0.85 (0.23)		0.73 (0.16)	
Education Status^*^							
Low Literacy	113	0.96 (0.14)	0.34	0.85 (0.30)	0.89	0.80 (0.28)	0.81
High School	26	0.93 (0.12)		0.88 (0.23)		0.78 (0.18)	
University	11	0.90 (0.27)		0.85 (0.20)		0.75 (0.25)	
Living Place							
Urban	138	0.95 (0.15)	0.70	0.85 (0.30)	1.00	0.80 (0.29)	1.00
Rural	12	0.92 (0.26)		0.85 (0.17)		0.80 (0.31)	

*: for these variables, we used the mean and SD in above tables.

IQR = interquartile range; SG= standard gamble; TTO = time trade off; VAT = visual analogue thermometer

#### Clinical Characteristics and Utility

As the obtained utility scores for diabetes types, 1 and 2 were almost identical, we report the results combined. Of the 150 studied patients, 90.7% had diabetes type 2; an equal percentage did not have any non-diabetic comorbidity, and 97 (64.7%) cases reported having other diabetic comorbidities. Non-diabetic comorbidities significantly affected HRQoL with the SG and VAT techniques, but utility values with the TTO analysis were significantly affected by other diabetic comorbidities. For example, DR patients who suffered from mild to moderate cancer overall reported an additional 0.01 and 0.06 loss in their HRQoL based on SG and TTO methods, respectively. In 66 cases, (44%) there was some degree of vision impairment in their better-seeing eye that insignificantly imposed some additional disutility.

Smoking significantly impacted utility scores derived with the SG and TTO methods, but we observed no significant correlation with the VAT method. Overall, 22.7% of the participants were current or past smokers. The higher the BMI, the higher the imposed disutility on HRQoL, but these differences were significant only with the VAT method and when disutility was less than 0.01% (Table 3).

**Table 3:** Total health/death-utility for all untreated diabetic retinopathy by clinical factors

	**No**	**SG Utility**		**TTO Utility**		**VAT Utility**	
		**Median (IQR)**	***P*-value**	**Median (IQR)**	***P*-value**	**Median (IQR)**	***P*-value**
Diabetes types							
1	14	0.97 (0.10)	0.32	0.82 (0.32)	0.65	0.76 (0.19)	0.49
2	136	0.94 (0.15)		0.86 (0.30)		0.80 (0.30)	
Non-diabetic Comorbidity							
No	136	0.95 (0.16)	0.00	0.85 (0.30)	0.35	0.80 (0.28)	0.05
Cancer	5	0.94 (0.09)		0.75 (0.20)		0.74 (0.20)	
Heart failure	9	0.70 (0.26)		0.97 (0.15)		0.56 (0.40)	
Diabetic Comorbidity							
0	53	0.96 (0.15)	0.44	0.99 (0.20)	0.00	0.80 (0.30)	0.31
≥ **1**	97	0.94 (0.15)		0.80 (0.20)		0.75 (0.27)	
Vision impairment (Better eye)^*^							
No	84	0.89 (0.16)	0.29	0.85 (0.19)	0.39	0.81 (0.22)	0.32
Yes	66	0.86 (0.18)		0.82 (0.23)		0.77 (0.26)	
Current/past Smoker							
No	116	0.96 (0.14)	0.06	0.90 (0.21)	0.00	0.80 (0.28)	0.60
Yes	34	0.91 (0.13)		0.70 (0.19)		0.77 (0.30)	
BMI							
Underweight/ Normal	101	0.94 (0.14)	0.74	0.85 (0.27)	0.64	0.80 (0.25)	0.01
Overweight	37	0.96 (0.15)		0.85 (0.30)		0.75 (0.27)	
Obese	12	0.93 (0.20)		0.77 (0.32)		0.55 (0.29)	

*: for these variables, we used the mean and SD in above tables.

BMI = body mass index; SG= standard gamble; TTO = time trade off; VAT = visual analogue thermometer.

Since diabetic or non-diabetic comorbidity in earlier analyses significantly impacted DR utility, we adjusted results accordingly to apply a significance level of 0.01. In all scenarios, the severity of disease was the main determinant of HRQoL such that with increasing severity, the disease disutility exponentially amplified, and all were significant at a significance level of 0.01. Higher DR utility scores correlated with unadjusted policy and vision health scales with the SG approach without any disutility, while lowest ones related to unadjusted policy scale with the VAT method and 0.50 disutility. The observed utility differences between these two scales were similar for adjusted and unadjusted comorbidities, utility derivation methods, and 6 defined vasculopathy with maculopathy states; all statistically significant at a 0.01 level. Results show that having maculopathy in any of the three studied DR states significantly imposed additional disutility. For instance, based on the TTO approach, DR patients in the proliferative state with DME, on average incur 9% more disutility compared with their counterparts without DME (Table 4).

**Table 4:** Adjusted and unadjusted utility values of diabetic retinopathy states with the utility assessment methods based on untreated visual acuity in the better seeing eye

**Health Scales**	**Non-diabetic Comorbidity-adjusted**	**Methods**	**No DR to Moderate NPDR (37)**	**Severe NPDR (29)**	**DR Stages (Number)**		**Severe NPDR (23)**	**PDR (18)**	***P*-value**	***P*-value**
				**Without DME**	**PDR (22)**	**No DR to Moderate NPDR (21)**	**With DME**			
					Median (IQR) Utility				0.00	<0.01
PS	Unadjusted	SG	1.00 (0.00)	0.96 (0.05)	0.90 (0.12)	1.00 (0.04)	0.92 (0.09)	0.84 (0.14)		
		TTO	0.95 (0.02)	0.90 (0.10)	0.75 (0.16)	0.90 (0.07)	0.80 (0.14)	0.65 (0.18)	0.00	
		VAT	0.95 (0.04)	0.85 (0.08)	0.70 (0.18)	0.85 (0.10)	0.70 (0.19)	0.58 (0.22)	0.00	
	Adjusted	SG	1.00	0.96	0.92	0.98	0.93	0.87	0.00	<0.01
		TTO	0.95	0.89	0.74	0.91	0.82	0.67	0.00	
		VAT	0.95	0.87	0.70	0.87	0.72	0.60	0.00	
VTS	Unadjusted	SG	1.00 (0.00)	0.96 (0.04)	0.92 (0.10)	1.00 (0.00)	0.93 (0.08)	0.86 (0.13)	0.00	<0.01
		TTO	0.97 (0.02)	0.90 (0.09)	0.78 (0.12)	0.93 (0.05)	0.83 (0.12)	0.69 (0.14)	0.00	
		VAT	0.97 (0.01)	0.90 (0.10)	0.74 (0.15)	0.88 (0.04)	0.75 (0.15)	0.65 (0.19)	0.00	
	Adjusted	SG	1.00	0.97	0.92	1.00	0.94	0.87	0.00	<0.01
		TTO	0.98	0.90	0.81	0.95	0.85	0.72	0.00	
		VAT	0.97	0.92	0.75	0.90	0.79	0.68	0.00	

DME = diabetic macular edema; DR = diabetic retinopathy; NPDR = non-proliferative diabetic retinopathy; PDR = proliferative diabetic retinopathy; PS = policy scale; SG= standard gamble; TTO = time trade off; VAT = visual analogue thermometer; VTS= vision truncatedscale.

##### Sensitivity Analysis

DR utility values in the absence of DME vary between 1.00–0.92 in none to mild DR, 0.92–0.70 in severe NPDR and 0.59–0.78 in PDR states. In the presence of DME, these ranges were 1.00–0.83, 0.84–0.60 and 0.74–0.50, respectively. Average scores for all patients in any method varies between 0.95 (IQR 0.03) to 0.80 (IQR 0.30). In all scenarios, values were highest with standard gamble, lowest with the VAT, and TTO values were in between.

## Discussion

Most studies investigating the utility for ophthalmic diseases have been conducted in a few high-income countries (20–24), and some of them have used the indirect multi-attribute measurement tools such as the EQ-5D, SF-6D, and Quality of Well-Being Index (QWB) (25, 16), However, the sensitivity of the mentioned indices to assess the impact of different diseases such as ophthalmic disorders is uncertain. (260 To the best of our knowledge, this is the first time DR is addressed using direct preference-based subjective utility valuation methods in a middle-income country.

We measured the impact of DR on the HRQoL in 150 Iranians with diabetes based on patient perception of their HRQoL using both policy and perfect vision health scales in a different cultural atmosphere compared to the developed world.

In addition to the severity of DR, according to the TTO method, factors that significantly affected studied patients were gender, smoking status, and diabetic comorbidity, but with the SG approach, the only determinant was non-diabetic comorbidity at a 0.05 level of significance.

Mean perfect health/death TTO utility in hospital-based studies by Brown et al. and Sharma et al. were 0.77 and 0.79, respectively (20, 24), while the adjusted TTO score in another community-based study was 0.92 (15). These values reached 0.78, 0.73 and 0.87, respectively, for moderate DR in the mentioned studies. In addition, Shah et al. have reported a perfect vision health/double blindness scale TTO score as high as 0.88 (22). Mean SG utility score for DR was 0.87–0.88 in three published comparable hospital-based studies (20, 21, and 23). This score based on perfect vision health policy in the study by Lee et al. was 0.79. Therefore, our participants reported higher SG and slightly less TTO utility scores compared with their Western counterparts. These differences possibly arise from three sources. Firstly, it seems that Iranians, owing to their sociocultural setting, are more risk aversion compared with people of developed countries. They also have stronger religious and conservative convictions so that many of them believe death is predetermined by the God’s will and not modifiable by human interventions. In fact, some found it offensive to discuss trading off life years or accepting the risk of death because their religious beliefs did not allow them to think of giving up their lives. Thus spiritual beliefs may influence patient’s health preferences in that giving up life-years is not a choice for some of them (27).

Lee et al. reported the SG utility scores for five common ophthalmic diseases; DR had the lowest disutility compared with cataract (0.91), refractive errors (0.89), glaucoma (0.88), and macular degeneration (0.84) (21). In Iran, an unpublished paper by Alinia et al. obtained a 0.98 the standard gable score for refractive errors, which is higher than DR utility scores.

The impact of ophthalmic diseases on the QoL is comparable with other chronic diseases. TTO-based disutility of DR is higher than chronic obstructive pulmonary diseases (median: 0.95) (28), treated arteriosclerosis obliterans with primary stent placement (mean: 0.91) (29), treated colorectal cancer in mild to moderate stages (mean: 0.90) (30), mild angina (mean: 0.90) (31) and asthma (mean: 0.89) (32), but is lower than other chronic and non-communicable diseases such as rheumatoid arthritis (median: 0.77) (33), treated severe colorectal cancer (mean: 0.71) (30), moderate angina (mean: 0.70), severe angina (mean: 050) and severe depression (mean: 0.45) (31).

Aside from patient’s sociodemographic and clinical characteristics, the methodological specification was a major determinant in final resulted utilities as well. These features included health scales, adjustment and utility elicitation methods that will be discussed below.

### 

#### Patient and Methodological Characteristics and DR Utility

On average, our patients with DR were willing to trade off 15% of their remaining time and accepted only 5% death risk in return for perfect health, whereas, the 80% score on their overall health status means that they believe 20% loss of their health status is due to DR. In terms of sociodemographic factors, only the TTO technique showed a significant effect on HRQoL, and the highest disutility was imposed on females. This subgroup lost more than 20% of their QoL to DR while this figure was less than 11% for males. As for other socioeconomic factors such as age, place of residence, education status, and economic status, lack of relationships is not for certain, because the sample size of at least one of their subgroups is less than 12, and increasing the number of these participants in each subgroup could lead to finding significant correlations.

DR poses limitations on patients in three dimensions: physical, emotional, and social. Physical restrictions for studied patients encompass daily routines such as walking, sports, driving, reading, and other personal affairs. Emotional constraints include frustration, fear of further vision loss and becoming blind in the future, concern about the future of their family, and loss of self-esteem. Finally, the social limitations of DR include fear of losing social opportunities, relationships, responsibilities and also recreation, social isolation and so on (34, 35). As DR becomes more severe, more restrictions will be imposed on the patients, and thus their QoL would further decrease. In the Iranian culture, women bear a considerable proportion of domestic and family responsibilities; therefore, on one hand, their disabilities cause greater limitations and disutility compared with men, and on the other hand, DR-attributed restrictions affect women’s ability to afford living facilities and limits their work and marital opportunities. Therefore, these groups of patients endure higher disutility.

Further changes of HRQoL, based on DR states, is the function of increasing returns, meaning that loss of HRQoL with growing DR severity will increase exponentially.

#### DR, Vasculopathy, and Maculopathy

According to the adjusted SG, in none to moderate NPDR, severe NPDR, and PDR stages, respectively 100%, 43.0%, and 39.0% of the incurred DR disutility was attributed to macular edema. These figures were respectively 44.5%, 39.0%, and 21.2% based on the adjusted TTO and 61.5%, 53.6%, and 25.0% with the VAT method. All analyses indicated that macular edema contributed a significantly greater share to the imposed disutility in early stages, which gradually decreased in more advanced stages. Macular edema directly causes visual acuity loss and decreases the patient’s HRQoL and vasculopathy in higher stages intensify its effects.

Regarding this definition, we can consider 16 (4-by-4) scenarios for DR-related QoL that are listed in Table 5. Any DR patient could experience at least one of these potential combined states of vasculopathy and maculopathy and bear a different disutility. This classification is novel and as the previous studies did not take this differential combined approach, we cannot directly compare our findings with them.

**Table 5:** Scenarios for diabetic retinopathy related quality of life.

**Diabetic Retinopathy**	**1**	**2**	**3**	**4**
Vasculopathy	≤ Moderate Non-Proliferative DR	Severe Non-Proliferative DR	Proliferative DR	Complicated Proliferative DR
Maculopathy	≤ Dry	Focal Edema Fovea Spared	Focal Edema Fovea Involved	Diffuse macular edema

DR = diabetic retinopathy.

SG method did not have enough sensitivity for DR severity because of participants’ varied demographics such as education level, age and religious beliefs, which makes it unsuitable for applying. This is while the TTO, due to its simplicity rather than SG and being preference-based, has shown to be valid, reliable, and sensitive to DR stages. Therefore, we recommend the TTO method for measuring utility scores in adult patients with ophthalmic disorders in Iranian settings and eastern cultures.

As demonstrated, for similar states of DR disorder, the policy scale showed overall higher disutility compared with the perfect vision scale. The perfect vision scale is a truncated policy scale; this means that identical values on these scales do not indicate the same disease effect on patients’ QoL. Kymes et al. refer to the perfect vision scale as vision truncated scale (VTS) (5).

#### Study Strengths and Limitations

The current study has several strengths. Firstly, compared with most related studies, we used adjusted and unadjusted three utility assessment methods as well as two scales; this means 12 scenarios, which show us the importance of method selection in cost-utility analysis and its impact on resulting resource allocation (36). Secondly, both types of diabetic patients were included in this investigation, although we did not see any significant HRQoL difference between them. Thirdly, we had enough samples in each health state with sufficient power to ensure the internal validity and their obtained significant differences. Fourthly, we did not eliminate the 7 disabled patients or those who could not speak properly, and we interviewed their first degree relatives. Fifthly, we have introduced a new definition of DR health states based on patient perception of QoL for measuring their health state utility.

Nonetheless, the current study is not free of limitations. It was not community-based, and participants were consecutively selected at one referral subspecialty hospital, so measured utilities may not be generalizable to all DR patients across the country. In addition, although we adjusted the utility to separate ophthalmic and nonophthalmic utility loss of diabetes multiorgan disease, in addition to socioeconomic factors, coexisting diabetic and non-diabetic comorbidities could confound the pure DR utility. Considering any comorbidity in the final utility suggests a higher possibility of being cost-effective for DR preventive or therapeutic interventions, and this means inefficient resource allocation.

In the current study, our sample size was not sufficient to look into severely impaired visual levels such as count finger, hand motion, light perception, no light perception, and blind cases or to separate macular edema state based on its two manifestations: diffusion and focal. These types of macular edema owing to their different effects on visual acuity and patient’s daily routines can have various impacts on the HRQoL. Therefore, we recommend investigators to consider these perspectives and issues in future studies.

## Conclusion

Utility scores in DR-experienced Iranian patients were highest with the standard gamble and lowest with the VAT method. The proffered utility elicitation method in Iranian patient populations could be the TTO approach, which should be used for policy-making along with other related economic evaluations. The novel model we employed for DR takes the effects of DME and vascularization into account separately and can provide a better estimate of the QoL for these patients.

## Ethical issues

The current study was certified by Human Research Ethics Committee of Tehran University of Medical Sciences and was accordance with the 1964 Helsinki declaration and its later amendments or comparable ethical standards. In addition to nature of the study was explained to the patients and enrollees gave their informed consent for participation.

Ethical issues (Including plagiarism, informed consent, misconduct, data fabrication and/or falsification, double publication and/or submission, redundancy, etc.) have been completely observed by the authors.
